# Flame-Retardant Systems Based on Chitosan and Its Derivatives: State of the Art and Perspectives

**DOI:** 10.3390/molecules25184046

**Published:** 2020-09-04

**Authors:** Giulio Malucelli

**Affiliations:** Department of Applied Science and Technology, and Local INSTM Unit, Politecnico di Torino, Viale Teresa Michel 5, 15121 Alessandria, Italy; giulio.malucelli@polito.it; Tel.: +39-0131-229369

**Keywords:** chitin, chitosan, flame retardance, bulky polymers, fabrics, textile materials, foams, flame spread tests, cone calorimetry, multifunctionality

## Abstract

During the last decade, the utilization of chitin, and in par0ticular its deacetylated form, i.e., chitosan, for flame retardant purposes, has represented quite a novel and interesting application, very far from the established uses of this bio-sourced material. In this context, chitosan is a carbon source that can be successfully exploited, often in combination with intumescent products, in order to provide different polymer systems (namely, bulky materials, fabrics and foams) with high flame retardant (FR) features. Besides, this specific use of chitosan in flame retardance is well suited to a green and sustainable approach. This review aims to summarize the recent advances concerning the utilization of chitosan as a key component in the design of efficient flame retardant systems for different polymeric materials.

## 1. Introduction

Polymeric materials, including bulk polymers, textiles and foams, if not inherently flame retarded will easily ignite and vigorously burn when exposed to an irradiative heat flux or to a flame. This behavior represents a dramatic drawback of such materials in all the uses that strictly demand fire-resistance [1,2].

In order to overcome this limitation, from the beginning of the fifth decade of the 19th century onwards, both academics and the industrial world started to design, produce and successfully employ the so-called flame retardants (FRs), i.e., special products designed to limit the propagation of a flame or even to prevent the ignition of the material into which they are incorporated or onto which they are deposited. As nicely described in the literature [3,4,5,6], upon the application of a flame or exposure to an irradiative heat flux, these additives may exploit different mechanisms:

- Char formation: in this case, FRs act in the condensed phase, promoting the formation of a stable carbonaceous residue on the surface (i.e., the so-called char), which acts as a barrier to inhibit gaseous products from diffusing to the flame, and protects the polymer’s surface from heat and air [7];
- Intumescence: upon activation, FRs swell together with the degrading material, leading to the formation of a porous foamed structure, which acts as a barrier to heat, air and pyrolysis products [8,9,10,11,12];
- Reactions in the gas phase: vapor-phase flame retardants are able to interfere with the free radical reactions involved in flame propagation [13,14];
- Cooling (heat sink mechanism): cooling occurs when the flame retardant decomposes endothermically to cool the pyrolysis zone located at the combustion surface [15];
- Dilution: this may occur in either the condensed or the gas phase. In the former, FRs act to dilute the polymer and reduce the concentration of decomposition-derived flammable gases. In the latter, FRs, upon activation, decompose to inert gases, which dilute the flammable gases.

In this context, different flame retardant systems have been developed. The first and most efficient FRs were halogen-based (mainly brominated and chlorinated) products; though they are still employed, some of them (namely, polychlorinated biphenyls, pentabromodiphenyl or decabromodiphenyl ethers) were recently banned by the USA and the EU, because of their high toxicity for human beings and animals [16,17,18]. Then, the research addressed the design of halogen-free flame retardant products, mainly containing phosphorus- or phosphorus/nitrogen compounds [12,19]. In fact, these types of FRs show good performances in different types of materials (bulky polymers, textiles and foams), and currently represent the best compromise between low environmental impact and effectiveness.

Quite recently, during the last 10 years, the research carried out in fire retardance has clearly demonstrated that some biomacromolecules and bio-sourced products, with peculiar chemical compositions and structures, can be exploited for the design of “green” and effective flame retardants for different types of materials. In particular, the academic research has identified some proteins (namely, whey proteins, caseins and hydrophobins), nucleic acids and natural extracts (such as phytic acid, tannins, banana pseudostem sap and pomegranate rind extract, among others) as potential components of flame retardant formulations [20,21,22].

**Figure 1:**
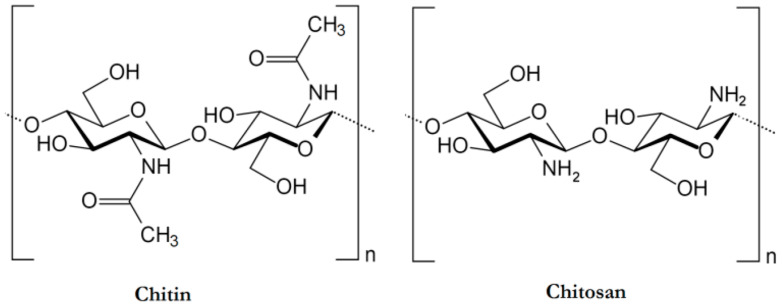
Structure of chitin (poly(b-(1-4)-*N*-acetyl-d-glucosamine)) and deacetylated chitin (chitosan). Reprinted from [27] under CC BY 4.0 license.

In this context, the possibility of exploiting chitosan and some of its derivatives in flame retardant recipes has been widely assessed; it is well known that chitosan, consisting of β-(1–4)-linked d-glucosamine and N-acetyl-d-glucosamine randomly distributed within the polymer (Figure 1), can be obtained by the partial deacetylation, performed in alkaline conditions, of chitin (i.e., poly(b-(1-4)-*N*-acetyl-d-glucosamine)), a key polysaccharide and the second most important natural polymer in the world after cellulose, mainly extracted from marine crustaceans, crab and shrimp shells [23,24]. Besides, in nature, chitin is structured in crystalline microfibrils that give rise to the structural components in the cell walls of yeast and fungi, as well as in the exoskeleton of arthropods. As a consequence, it shows very good mechanical features [25].

If the deacetylation degree of chitin reaches around 50%, the obtained chitosan can be dissolved into aqueous acidic media, thus converting the insoluble polysaccharide into a polyeletrolyte. This finding is ascribed to the protonation of the amino groups located at the C-2 position of the D-glucosamine repeat unit, and justifies the possible use of chitosan for the buildup of flame retardant Layer-by-Layer coatings on different types of fabrics, as discussed later.

Apart from the well-known applications (such as in the biomedical and pharmaceutical sectors, for food and nutrition, wastewater treatments and textile finishing, among others [26]), the chemical structure of chitosan suggests the possibility of using it as a carbon and nitrogen source in the design of low environmental impact flame retardant formulations; besides, the presence of such reactive groups as -NH_2_ and -OH can be successfully exploited in chemically modifying the biomacromolecule, thus introducing phosphorus-containing functionalities. This way, it is possible either to combine chitosan with other additives, in order to design high-performing flame retardants, or to obtain some derivatives that could be directly exploited for the design of effective flame retardant formulations.

It is worthy of note that chitosan alone, although not intrinsically flame retardant, is a very good carbon source that, if appropriately applied to a polymer substrate (i.e., bulky polymer, textile, foam, wood), can give rise to the formation of a stable char; besides, as explained in the following paragraphs, it can be successfully exploited, in combination with flame retardant additives, for the design of effective flame retardant systems, suitable for different polymer substrates.

This review work aims at describing the very recent progress concerning the use of chitosan and some of its derivatives in the design of efficient FRs for different types of flammable materials, i.e., bulk polymers, textiles and foams. Apart from flame retardance, the possible multifunctionality provided by these bio-sourced macromolecules will also be highlighted.

Finally, some perspectives related to further developments in the exploitation of this biomacromolecule as a low environmental impact component of flame retardant recipes will be discussed.

## 2. Thermal Stability of Chitosan as Assessed by Thermogravimetric Analyses

Before considering the effects of chitosan and/or its derivatives on the flame retardant behavior of different polymer substrates, it is worth summarizing the thermal behavior of the biomacromolecule as assessed by thermogravimetric analyses. In nitrogen, chitosan thermal degradation involves two main degradation steps: the first one takes place between 30 and 110 °C, and is attributed to the evaporation of the residual water. The second degradation step, which occurs between about 200 and 340 °C, is ascribed to the decomposition of the polymer chains; the overall weight loss after the second step is about 50%, though it may slightly change according to the deacetylation degree and average molecular weight [28].

## 3. Chitosan and Its Derivatives as Flame Retardants for Bulk Polymers

For the above-mentioned reasons, chitosan and its derivatives have largely been exploited in designing flame retardant recipes for bulk polymers. In the following, the main outcomes over the last 3 years will be thoroughly described. Chitosan has been either combined with other flame retardant additives, thus designing very effective FR systems, or has been chemically modified on purpose. Regarding the latter, Table 1 collects the main chemical modifications of chitosan done to provide flame retardant features to bulky polymers, which will be hereinafter summarized.

**Figure 2:**
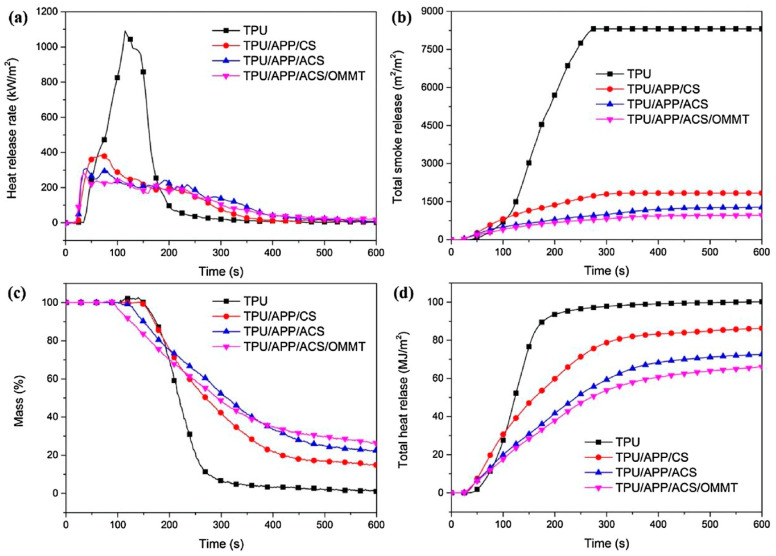
HRR—Heat Release Rate (**a**), THR—Total Heat Release (**b**), Mass loss (**c**) and TSR—Total Smoke Release (**d**) curves of the investigated systems. TPU: thermoplastic polyurethane; TPU/APP/CS: thermoplastic polyurethane containing 5 wt. % of ammonium polyphosphate and 5 wt. % of chitosan; TPU/APP/ACS: thermoplastic polyurethane containing 5 wt. % of ammonium polyphosphate and 5 wt. % of chitosan crosslinked with bis-(4-formylphenyl)-phenyl-phosphonate; TPU/APP/ACS/OMMT: thermoplastic polyurethane containing 4.5 wt. % of ammonium polyphosphate, 4.5 wt. % of chitosan crosslinked with bis-(4-formylphenyl)-phenyl-phosphonate and 1 wt. % of Cloisite 30B, an organo-modified montmorillonite. Reprinted with permission from [29]. Copyright 2018, Elsevier.

Zhang and co-workers crosslinked chitosan with bis-(4-formylphenyl)-phenyl-phosphonate; the resulting product was combined with ammonium polyphosphate and an organo-modified montmorillonite (namely, Cloisite 30B^®^) and incorporated into a thermoplastic polyurethane by melt mixing [29]. At 10 wt. % FR loading, the composites achieved self-extinction in vertical flame spread tests, showing, at the same time, a remarkable increase in the limiting oxygen index (29.0% vs. 20.8% for filled and unfilled thermoplastic polyurethane, respectively). Besides this, as assessed by forced combustion tests (irradiative heat flux: 50 kW/m^2^, Figure 2), the flame retardant recipe allowed for reducing the total heat release (about −34%), the peak of the Heat Release Rate (about −74%), and the Total Smoke Release (about −88%). All these findings were ascribed to the formation of a stable protective char, thanks to the concurrent catalytic carbonization of the nanoclay and the dehydration reactions promoted by the conversion of phosphonate into phosphonic groups in the crosslinked chitosan.

**Figure 3:**
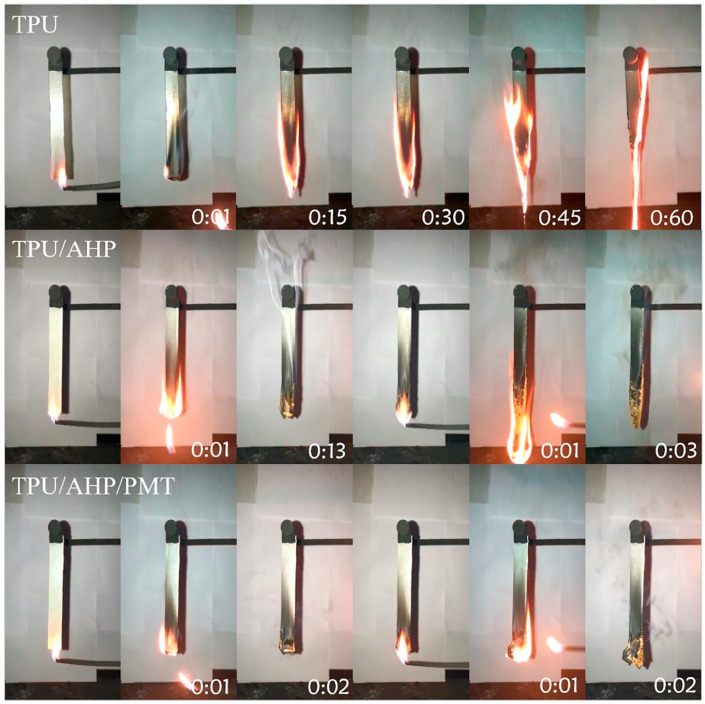
Video screenshots of TPU (thermoplastic polyurethane), TPU/AHP (thermoplastic polyurethane containing 10 wt. % of aluminum hypophosphite) and TPU/AHP/PMT (thermoplastic polyurethane containing 9 wt. % of aluminum hypophosphite and 1 wt. % of exfoliated montmorillonite decorated with phosphorylated chitosan) composites during UL-94 testing. Reprinted with permission from [30]. Copyright 2019, Elsevier.

Pursuing this strategy, Liu et al. [30] prepared montmorillonite nanosheets modified with phosphorylated chitosan, using an ultrasonic exfoliation methodology; then, the modified nanosheets were incorporated into a thermoplastic polyurethane (TPU) together with aluminum hypophosphite, by means of melt compounding performed in a micro twin-screw extruder. The formulation containing the aluminum salt and the modified nanoclay showed very good fire performances, achieving a limiting oxygen index of 28% (vs. 21% for unfilled TPU) and self-extinction in vertical flame spread tests (Figure 3). Besides, as compared with neat TPU, this flame retardant system manifested a significant decrease in both the peak of the Heat Release Rate (about −82%) and Total Smoke Release (−89%) in forced combustion tests performed at 50 kW/m^2^, thus demonstrating the synergistic effects taking place between the FR fillers.

Chen and co-workers [33] exploited the Layer-by-Layer method [34,35] for functionalizing diatomite particles with bi-layered assemblies made of chitosan and ammonium polyphosphate; these modified particles were embedded into an unsaturated polyester resin. Assembling nine bilayers on diatomite allowed the achieving of a V-0 rating in vertical flame spread tests; besides, as assessed in forced combustion tests, both the peak of the Heat Release Rate and the Total Heat Release were reduced by 40.8% and 18.1%, respectively.

Hassan et al. [31] demonstrated the occurrence of synergism between melamine salt of chitosan phosphate and organo-modified montmorillonite on the thermal stability and flame retardance of linear low density polyethylene (LLDPE). In thermogravimetric analyses, unlike unfilled LLDPE, for which the residue at 700 °C was negligible, the incorporation of 1 wt. % of modified nanoclay and 30 wt. % of the chitosan derivative promoted a significant increase in the char formation (residue around 10 wt. %); besides, in vertical flame spread tests, this formulation achieved self-extinction, together with a V-0 classification. Finally, in cone calorimetry tests, the flame retarded polyolefin exhibited a remarkable decrease in the peak of the Heat Release Rate (−76%), Total Heat Release (−37%), CO and CO_2_ yields, and fire growth index.

**Figure 4:**
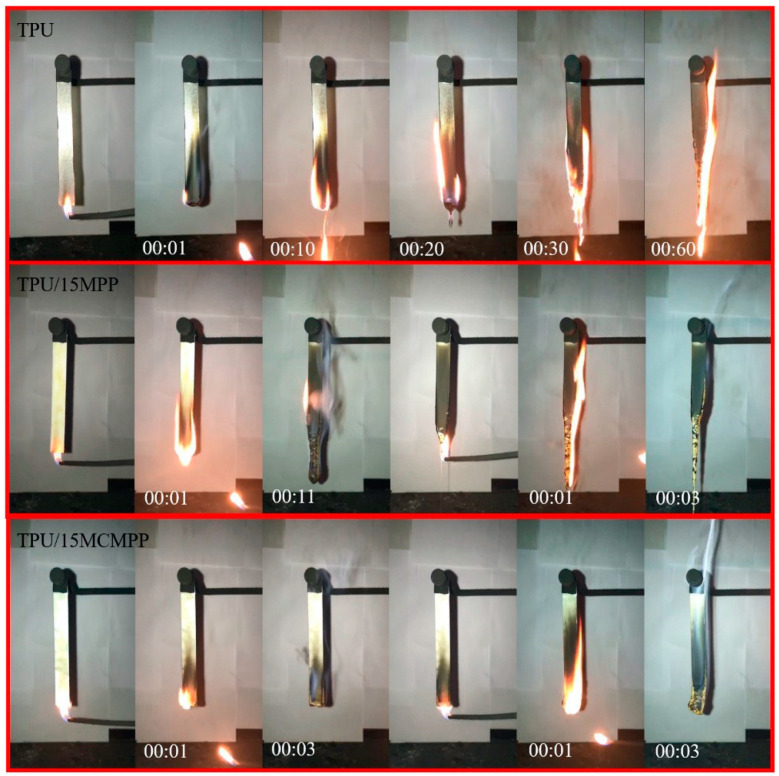
Video screenshots of TPU (thermoplastic polyurethane), TPU/MPP (thermoplastic polyurethane containing 15 wt. % of melamine polyphosphate) and TPU/MCMPP (thermoplastic polyurethane containing 15 wt. % of melamine polyphosphate microencapsulated by toluene diisocyanate crosslinked carboxymethyl chitosan) composites during UL-94 testing. Reprinted with permission from [32]. Copyright 2019, Elsevier.

Recently, melamine polyphosphate has been microencapsulated by toluene diisocyanate cross-linked carboxymethyl chitosan and then employed for preparing flame retardant thermoplastic polyurethane (TPU) composites [32]. In particular, the incorporation of 15 wt. % of the synthesized flame retardant was enough to achieve self-extinction in vertical flame spread tests (Figure 4) and a Limiting Oxygen Index value equal to 29.4% (unfilled polymer: 20.9%). Besides, as compared to the neat polymer matrix, the presence of the flame retardant was responsible for a significant decrease in the peak of the Heat Release Rate (−66%), the Total Heat Release (−24%), Total Smoke Release (−87%) and carbon monoxide production (−61%), thus clearly showing high fire performances.

**Figure 5:**
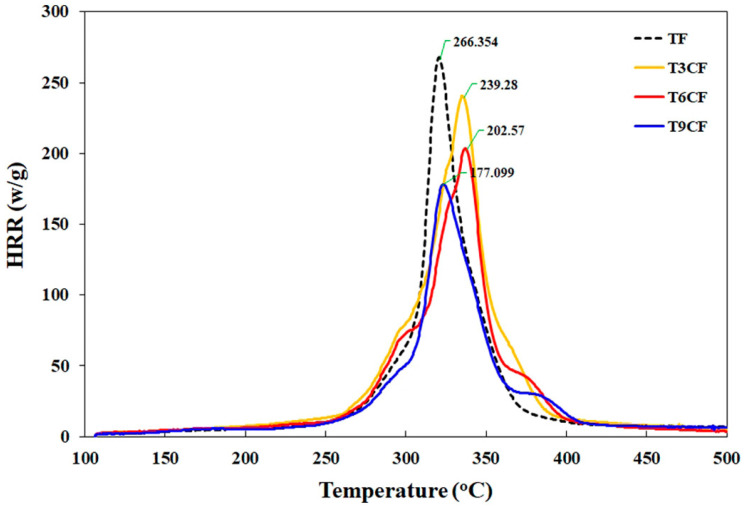
Heat Release Rate (HRR) vs. time curves for the different systems investigated: thermoplastic starch–flax fabric (TF), thermoplastic starch–chitosan–flax fabric (T*X*CF, where *X* indicates the weight % of chitosan). Reprinted with permission from [36]. Copyright 2018, Elsevier.

Prabhakar and Song [36] fabricated eco-friendly biocomposites combining thermoplastic starch, chitosan and flax fabrics. The effect of chitosan loading (between 3 and 9 wt. %) on the overall fire behavior of the biocomposites was assessed: in particular, 6 wt. % of chitosan was enough to achieve self-extinction in horizontal flame spread tests, a V0 classification in vertical flame spread tests and a Limiting Oxygen Index as high as 45.5% (vs. the 21.0% of thermoplastic starch). Besides, as assessed in pyrolysis-combustion flow calorimetry analyses, the Heat Release Rate was substantially decreased in the presence of increasing chitosan loadings (i.e., about −10%, −24% and −34% for the biocomposites containing 3, 6 and 9 wt. % of the biomacromolecule, respectively; Figure 5). These findings were attributed to the presence of chitosan in the biodegradable polymer matrix, which exhibited a high charring effect. Beside the flame retardance, chitosan improved the mechanical behavior of the composites, increasing the tensile strength and modulus while maintaining, at the same time, an acceptable elongation at break.

**Figure 6:**
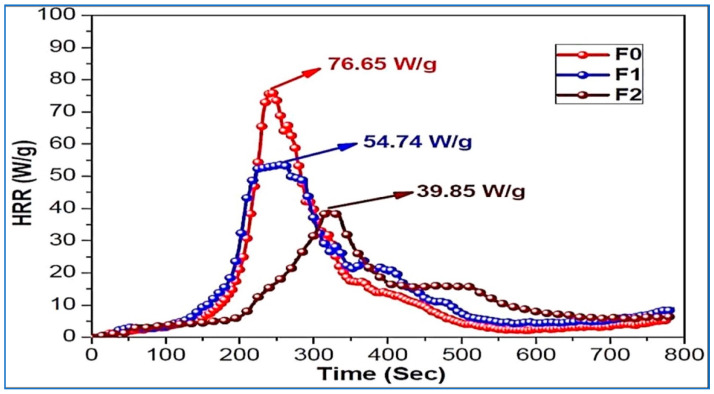
Heat release Rate (HRR) vs. time curves for the different systems investigated: F0 (without silica nanoparticles), F1 (with 2% *w*/*w* silica nanoparticles) and F2 (with 4% *w*/*w* silica nanoparticles). Reprinted with permission from [37]. Copyright 2020, Elsevier.

Very recently, silica nanoparticles at different loadings (namely, 2 and 4 wt. %) were exploited in combination with ammonium polyphosphate (30 wt. %) and chitosan (10 wt. %) for obtaining flame retarded polyurethane intumescent coatings [37]. As assessed by vertical flame spread tests, the flame retardant coatings, even those containing the lowest silica nanoparticle loadings, achieved self-extinction and a V-0 classification; besides, the Limiting Oxygen Index values of the FR intumescent coatings were as high as 30.5% and 35.7% for the systems embedding 2 and 4 wt. % of SiO_2_, respectively (LOI for unfilled polyurethane: about 24%). Finally, pyrolysis-combustion flow calorimetry tests showed a significant decrease in the peak of Heat Release Rate and total heat release (−48% for both) in the presence of 4 wt.% of silica, as compared to the unfilled polymer network (Figure 6).

**Figure 7:**
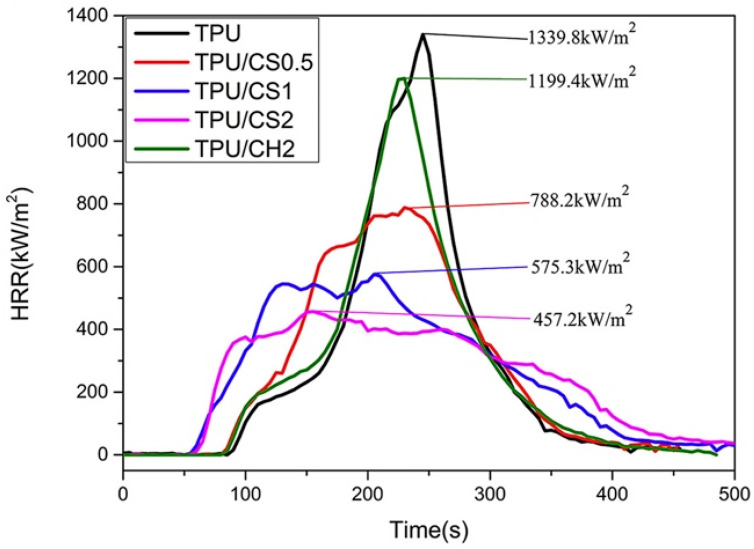
Heat Release Rate (HRR) vs. time curves of flame retardant thermoplastic polyurethane elastomer (TPU) composites. CH, chitin, CS, chitosan. Reprinted with permission from [38]. Copyright 2020, Wiley.

The flame retardant features provided by chitosan (acetylation degree: 90%) were recently investigated by Jiao and co-workers [38], who incorporated the biomacromolecule into a thermoplastic polyurethane elastomer using an internal mixer. This is the only example of using chitosan (without any chemical modification) alone in a bulky polymer. Even at 2 wt. % loading only, chitosan was able to remarkably reduce both the peak of Heat Release Rate −66%) and the Total Heat Release (−47%) with respect to the unfilled polymer matrix, as assessed by cone calorimetry tests (Figure 7). Besides, the amino groups of the biomacromolecule, by reacting with the decomposition compounds derived from the polyurethane thermoplastic elastomer, were capable of suppressing the smoke production.

## 4. Chitosan and Its Derivatives as Flame Retardants for Textiles

**Figure 8:**
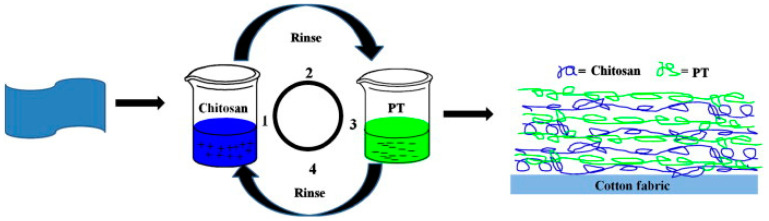
Scheme of the Layer-by-Layer process. PT = phosphorylated chitin. Reprinted with permission from [39]. Copyright 2015, Elsevier.

**Figure 9:**
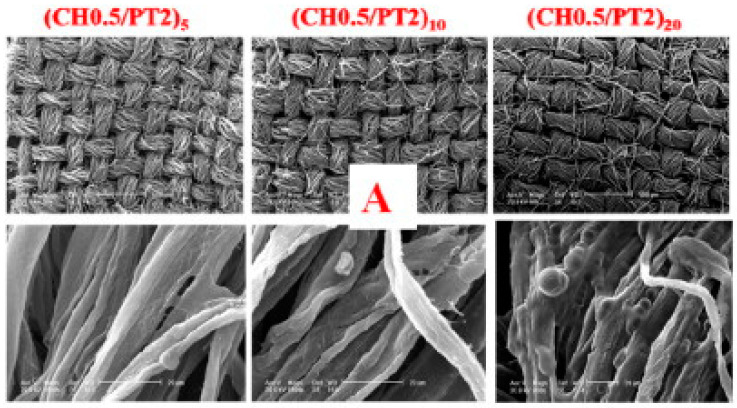
SEM images, at different magnification, of the residues of the LbL-treated fabrics after vertical flame spread tests. 5, 10 and 20 correspond to the number of deposited bi-layers. CH = chitosan; PT = phosphorylated chitin; 0.5 and 2 are the wt. % concentrations of the biomacromolecules in the dipping baths. Reprinted with permission from [39]. Copyright 2015, Elsevier.

Pan and co-workers [39] exploited the Layer-by-Layer method for depositing bi-layered assemblies consisting of chitosan and phosphorylated chitin on cotton fabrics (Figure 8); in particular, 5, 10 and 20 bi-layers were deposited. Vertical flame spread tests demonstrated that the assemblies consisting of 20 bi-layers prepared at a high phosphorylated chitin concentration (i.e., 2 wt. %) were able to provide self-extinction to the cellulosic substrate, keeping almost 90% of the fabric unburned. Besides, as assessed by cone calorimetry tests (performed at 35 kW/m^2^ irradiative heat flux), all the treated fabrics showed lower peaks of Heat Release Rate and Total Heat Release values with respect to their untreated counterparts. Finally, the intumescent character of the deposited assemblies was demonstrated by SEM analyses (Figure 9).

**Figure 10:**
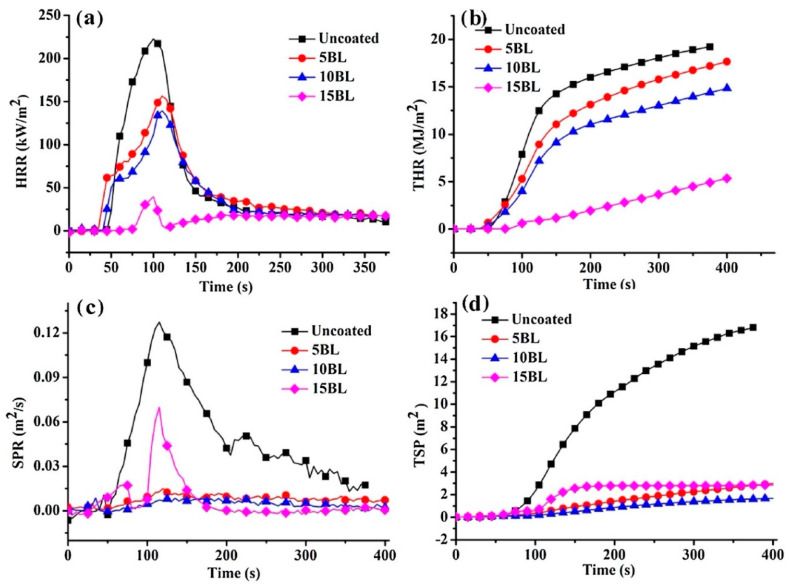
Results from cone calorimetry tests for untreated and LbL-treated cotton fabrics. Heat Release Rate—HRR (**a**), Total Heat Release—THR (**b**), Smoke Production Rate—SPR (**c**) and Total Smoke Production—TSP (**d**) curves. Reprinted with permission from [40]. Copyright 2018, Elsevier.

Liu et al. [40] exploited chitosan, sodium phytate and hydrolyzed (3-aminopropyl) triethoxysilane (APTES) for the design of LbL assemblies deposited on cotton fabrics. More specifically, the first bi-layer consisted of sodium phytate and hydrolyzed APTES; then, for the second bi-layer, APTES was replaced with chitosan. The so-obtained assemblies were made of 5, 10 and 15 bi-layers. The cellulosic substrate treated with 15 bi-layers achieved self-extinction in vertical flame spread tests, and its Limiting Oxygen Index was about 29% (untreated cotton: 18.1%). As clearly shown by cone calorimetry tests (performed at 35 kW/m^2^ irradiative heat flux, Figure 10), the combination of chitosan, sodium phytate and APTES significantly lowered the peak of the Heat Release Rate, the Total Heat Release, the Smoke Production Rate and the Total Smoke Production of the treated fabrics.

Sheikh and Bramhecha highlighted the multifunctional features (including flame retardance) that a two-stage chitosan-based finishing process can provide to linen fabrics; in particular, the fabrics were first treated with chitosan and citric acid, then with phytic acid and thiourea [41]. Apart from crease recovery, antibacterial activity and UV protection, the combination of the four components allowed the achieving of a Limiting Oxygen Index as high as 39.8%, which showed a decreasing trend, reaching 25% after 20 washing cycles, thus indicating the rather limited durability of the treated fabrics.

**Figure 11:**
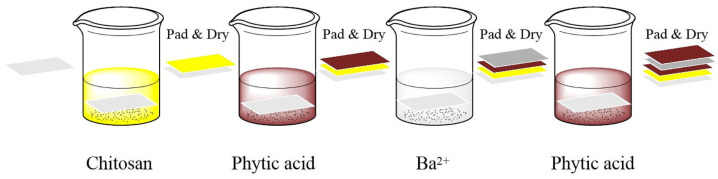
Coating process based on chitosan, phytic acid and barium ion by dip-pad-dry method. Reprinted with permission from [42]. Copyright 2019, Elsevier.

Zhang and co-workers [42] exploited a dip-pad-dry process (schematized in Figure 11) for depositing chitosan and phytic acid as an intumescent FR system, using Ba^2+^ as a synergist, on cotton fabrics.

**Figure 12:**
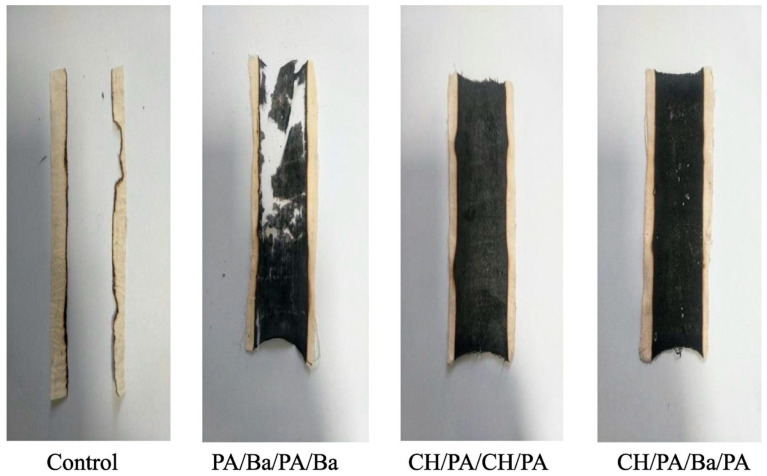
Residues at the end of the flame spread tests performed in a horizontal configuration. PA/Ba/PA/Ba: quad-layer made of phytic acid/Ba^2+^/phytic acid/Ba^2+^; CH/PA/CH/PA: quad-layer made of chitosan/phytic acid/chitosan/phytic acid; CH/PA/Ba/PA: quad-layer made of chitosan/phytic acid/Ba^2+^/phytic acid. Reprinted with permission from [42]. Copyright 2019, Elsevier.

As revealed by flame spread tests performed in a horizontal configuration, the treated fabrics did not achieve self-extinction, despite a remarkable increase in the residues at the end of the tests, which were dense and coherent, especially when chitosan was employed as a component of the deposited coating (Figure 12).

**Figure 13:**
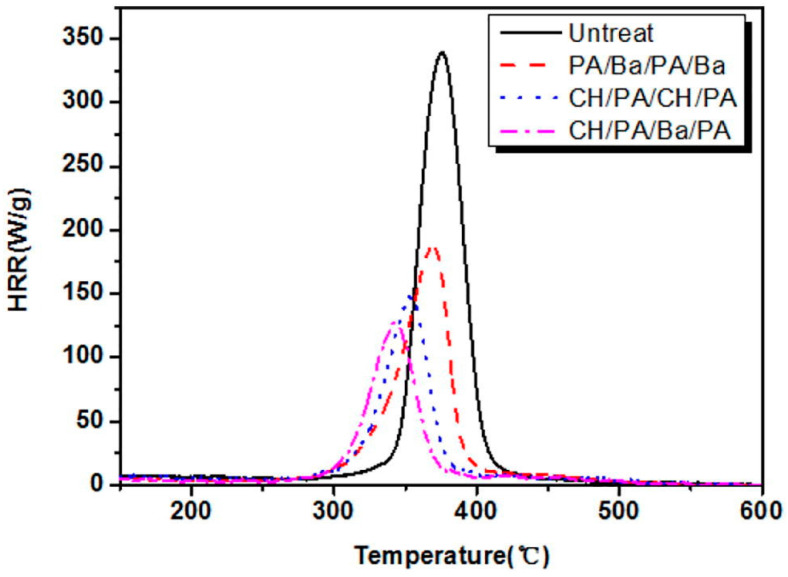
Results from pyrolysis-combustion flow calorimetry tests. PA/Ba/PA/Ba: quad-layer made of phytic acid/Ba^2+^/phytic acid/Ba^2+^; CH/PA/CH/PA: quad-layer made of chitosan/phytic acid/chitosan/phytic acid; CH/PA/Ba/PA: quad-layer made of chitosan/phytic acid/Ba^2+^/phytic acid. Reprinted with permission from [42]. Copyright 2019, Elsevier.

The deposited coatings showed enhanced flame retardant properties, as revealed by the significant decrease in the peak of the Heat Release Rate and the Total Heat Release values in the pyrolysis-combustion flow calorimetry tests (Figure 13).

**Figure 14:**
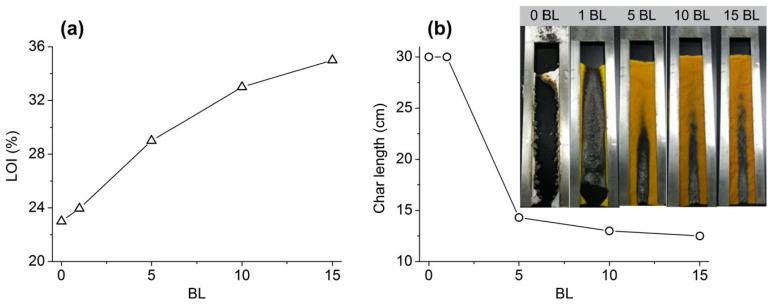
Results from LOI (**a**) and flame spread tests (**b**) for untreated and LbL-treated silk fabrics. Reprinted with permission from [43]. Copyright 2019, Elsevier.

Recently, Lv and coworkers exploited the Layer-by-Layer padding method for building up assemblies made of chitosan and vitamin B2 sodium phosphate on silk fabrics [43]. In particular, 1, 5, 10 and 15 bi-layers were successfully deposited on the protein substrate. As clearly demonstrated by flammability tests (i.e., Limiting Oxygen Index and vertical flame spread tests, Figure 14), increasing the number of deposited bi-layers improved the overall FR performances of the treated fabrics. In particular, 5 bi-layers were enough for achieving self-extinction and a Limiting Oxygen Index as high as 28.9% (vs. 23% for untreated silk). Finally, the multifunctionality provided by the LbL architectures was proven by performing antibacterial tests on the treated fabrics against *E. coli* and *S. aureus*; in all cases, the treated silk exhibited inhibition rates beyond 90%.

**Figure 15:**
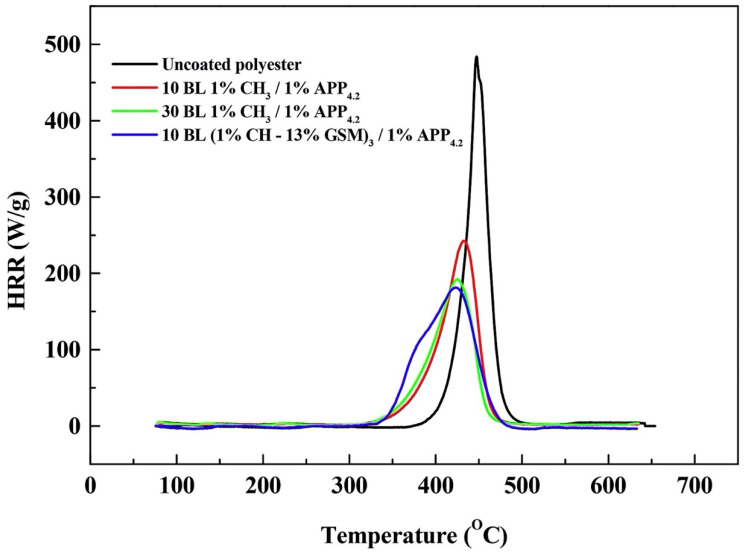
Results from pyrolysis-combustion flow calorimetry tests performed on untreated and LbL-treated polyester fabrics. 10 BL 1% CH_3_/1%APP_4.2_: assembly made of 10 bilayers of chitosan and ammonium polyphosphate; 30 BL 1% CH_3_/1%APP_4.2_: assembly made of 30 bilayers of chitosan and ammonium polyphosphate; 10 BL (1% CH-13% GSM)_3_/1%APP_4.2_: assembly made of 10 bilayers of chitosan and ammonium polyphosphate, containing guanidine sulfamate. Reprinted with permission from [44]. Copyright 2019, Elsevier.

Jordanov and co-workers [44] designed intumescent FR nanocoatings on polyester fabrics, using the dipping LbL approach. More specifically, they combined chitosan (positively charged) with ammonium polyphosphate (negatively charged) in bi-layered assemblies that were further modified by adding guanidine sulfamate, urea or thiourea to the aqueous solutions of chitosan. This way it was possible to assess the effect of these low-molecular weight nitrogen- and nitrogen/sulfur-based derivatives on the flame retardant properties of the LbL-treated polyester fabrics. 10, 25 and 30 bi-layers were deposited. Self-extinction was achieved only when 30 bilayers of chitosan/ammonium polyphosphate were deposited; conversely, the presence of 10 bilayers only comprising guanidine sulfamate allowed for achieving the same fire performances obtained with 30 bilayers, but without the low-molecular weight derivative. Furthermore, these results were confirmed by pyrolysis-combustion flow calorimetry tests (Figure 15), with a significant decrease (−61.7%) in the peak of the Heat Release Rate for the fabric treated with 10 bilayers incorporating guanidine sulfamate as compared to the untreated textile.

In a pioneering work, El-Tahlawy added chitosan (deacetylation degree: 90%) during the phosphorylation of cotton fabrics, aiming at designing green flame retarded cellulosic fabrics [45]. In particular, a chitosan/citric acid solution was mixed with a phosphorylation bath containing sodium hypophosphite, butanetetracarboxylic acid and diammonium hydrogen phosphate; the fabrics achieved a wet pick up between 90% and 93%, and were then dried (for 5 min at 85 °C) and finally cured (within 150 and 170 °C for 1–3 min). As revealed by vertical flame spread tests, the presence of chitosan, even at low concentrations, in the phosphorylation bath (i.e., 1 and 2 wt. %) was enough to provide the treated fabrics with self-extinction, significantly decreasing the char length. Besides, a 10 wt. % concentration of diammonium hydrogen phosphate provided the treated fabrics with a good washing resilience (up to 25 washing cycles, according to the AATCC test method).

Kundu and co-workers grafted phosphorylated chitosan onto polyamide 66 fabrics, which were then treated with (3-aminopropyl) triethoxysilane by means of a sol-gel process, aiming at designing a cross-linking coating [46]. As assessed through flame spread tests in a vertical configuration, the combination of grafting and sol-gel allowed for preventing the melt dripping phenomena; besides, cone calorimetry tests (performed at 35 kW/m^2^ irradiative heat flux) indicated a significant decrease (by 30%) in the peak of the Heat Release Rate with respect to the untreated fabric, ascribed to a joint effect between the sol-gel derived silica and the char-forming character of the phosphorylated chitosan.

**Figure 16:**
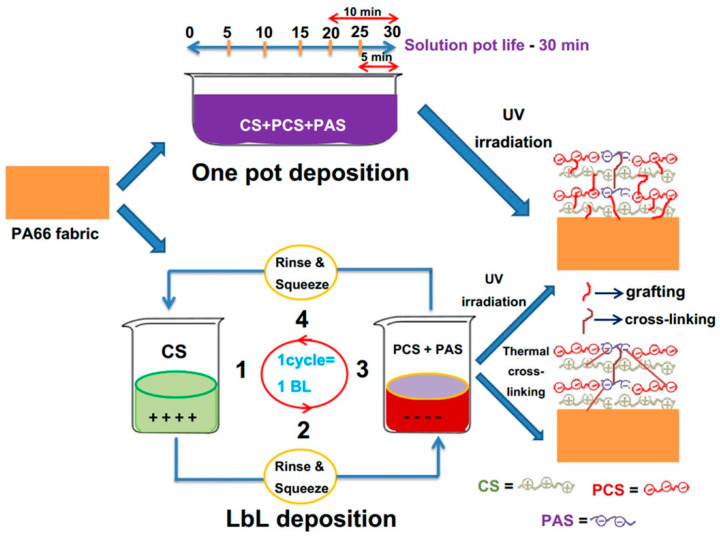
Scheme of the LbL vs. “one pot” deposition on polyamide 66 fabrics. CS = chitosan, PCS = phosphorylated chitosan, PAS = polyacrylate sodium, PA66 = polyamide 66. Reprinted with permission from [47]. Copyright 2020, Elsevier.

Pursuing this research, the same group [47] investigated the fire retardant behavior provided by chitosan and phosphorylated chitosan deposited on polyamide 66 fabrics together with polyacrylate sodium. In particular, a Layer-by-Layer approach was compared with a “one pot” deposition, whereby all the “one pot” and a part of the LbL-treated fabrics were exposed to UV radiation in order to promote grafting reactions among polyamide 66, polyacrylate sodium and chitosan chains; in addition, some LbL-treated fabrics underwent a low-temperature thermal treatment to trigger cross-linking reactions among the adjacent layer constituents (Figure 16).

Scanning electron microscopy analyses clearly showed a better coverage and distribution of the coatings derived via the Layer-by-Layer method with respect to the “one pot” deposition. This finding further supported the better FR performances found for the LbL-derived coatings as compared to their “one pot” counterparts, though both achieved V1 classification in vertical flame spread tests and suppressed the melt dripping of the synthetic fabric. Table 2 collects the results of cone calorimetry tests carried out at 35 kW/m^2^ irradiative heat flux: it is worthy of note that the LbL assemblies gave rise to the greatest decrease in the peak of the Heat Release Rate.

Pan and co-workers exploited the Layer-by-Layer method for designing a durable flame retardant coating on cotton fabrics [48]. To this aim, the latter were treated with polyethyleneimine (positively charged) and hypophosphorous acid-modified chitosan (negatively charged) bi-layers, and then subsequently crosslinked using genipin, a natural extract from gardenia fruit, before 5 or 10 bi-layers were deposited on the cellulosic substrate.

**Figure 17:**
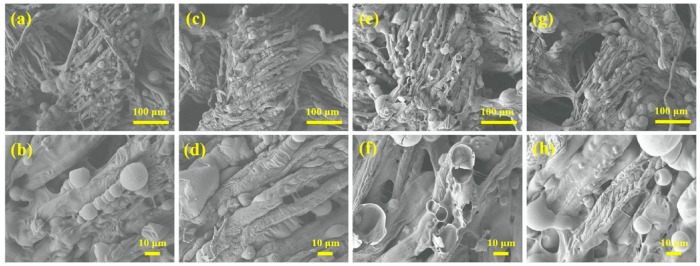
Scanning Electron Microscopy micrographs of the residues after horizontal flame spread tests. C-(PEI/HACH)5 (**a**,**b**): cotton treated with 5 bi-layers of polyethyleneimine; C-(PEI/HACH)5-crosslink (**c**,**d**): cotton treated with 5 bi-layers of polyethyleneimine and crosslinked with genipin; C-(PEI/HACH)10 (**e**,**f**): cotton treated with 10 bi-layers of polyethyleneimine; C-(PEI/HACH)10-crosslink (**g**,**h**): cotton treated with 10 bi-layers of polyethyleneimine and crosslinked with genipin. Reprinted with permission from [48]. Copyright 2019, Elsevier.

**Figure 18:**
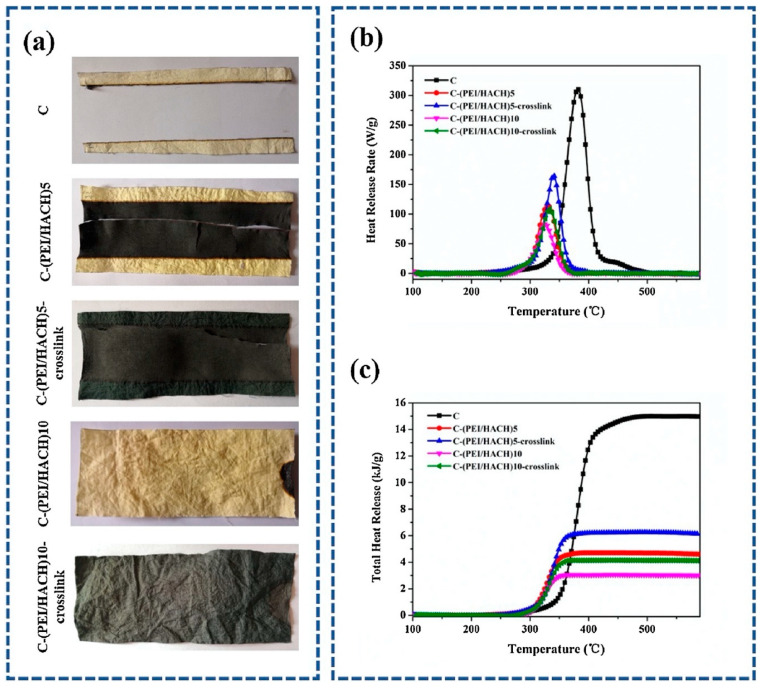
Photographs of untreated and treated cotton fabrics after horizontal burning tests (**a**). Heat Release Rate (**b**) and Total Heat Release (**c**) as a function of temperature for untreated and treated cotton fabrics. C-(PEI/HACH)5: cotton treated with 5 bi-layers of polyethyleneimine; C-(PEI/HACH)5-crosslink: cotton treated with 5 bi-layers of polyethyleneimine and crosslinked with genipin; C-(PEI/HACH)10: cotton treated with 10 bi-layers of polyethyleneimine; C-(PEI/HACH)10-crosslink: cotton treated with 10 bi-layers of polyethyleneimine and crosslinked with genipin. Reprinted with permission from [48]. Copyright 2019, Elsevier.

As assessed by flame spread tests performed in a horizontal configuration, 10 bi-layers, irrespective of the cross-linking with genipin, provided the fabrics with self-extinction; besides, the residues at the end of the tests confirmed the intumescent-like behavior of the designed assemblies (Figure 17). In addition, microcone calorimetry tests showed an outstanding decrease in both the peak of the Heat Release Rate (up to −73%) and the Total Heat Release values (up to −80%) of the fabrics treated with 10 bi-layers, compared to untreated cotton, as shown in Figure 18.

**Figure 19:**
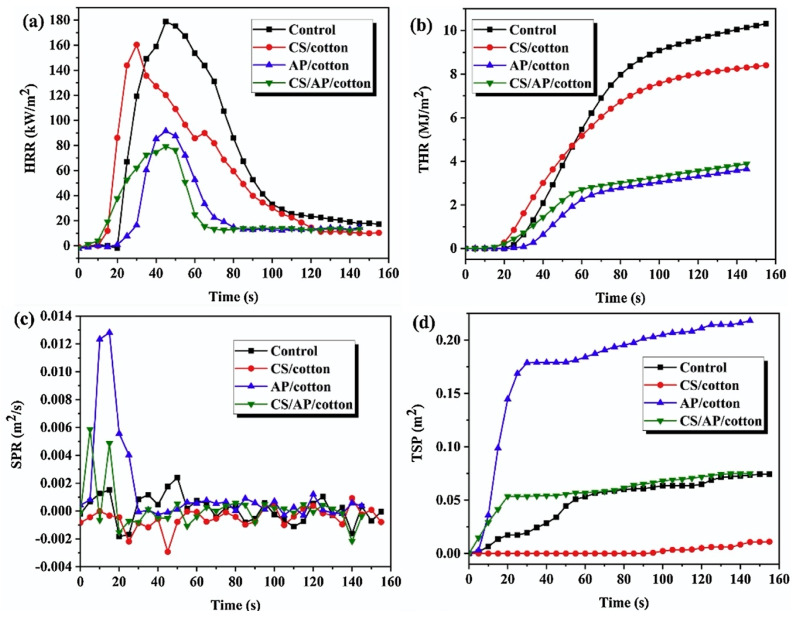
Results from cone calorimetry tests for untreated and treated cotton fabrics. Heat Release Rate—HRR (**a**), Total Heat Release—THR (**b**), Smoke Production Rate—SPR (**c**) and Total Smoke Production—TSP (**d**) curves. Reprinted with permission from [49]. Copyright 2020, Elsevier.

Very recently, a fully bio-based LbL coating was designed and applied to cotton fabrics, combining chitosan and ammonium phytate bi-layers, by Li and co-workers [49]. For comparison, the fabrics were also treated with chitosan or ammonium phytate only, achieving comparable add-ons (about 8%). Table 3 collects the main results from the flammability (i.e., Limiting Oxygen Index and vertical flame spread tests) and forced-combustion tests (Figure 19) for untreated cotton and the treated fabrics. It is worthy of note that, despite the fact that the results of the flammability tests for cotton treated with ammonium phytate only and for the fabric LbL-treated with both ammonium phytate and chitosan were almost comparable, the combination of the two components in the LbL assembly allowed for achieving the greatest decrease in the peak of the Heat Release Rate (about −56% as compared to the untreated cotton) and the highest level of final residues, hence highlighting the remarkable char-forming character of the designed LbL coating.

**Figure 20:**
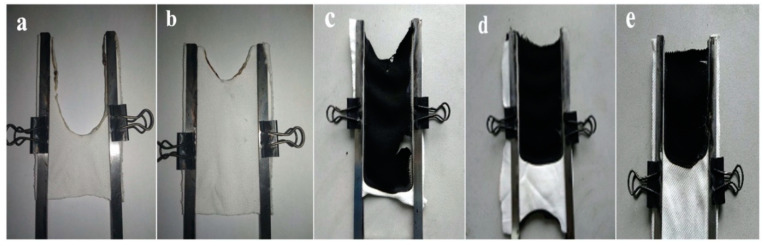
Digital images of uncoated and coated polyester fabrics after Limiting Oxygen Index testing. (**a**) uncoated polyester, (**b**) LbL-treated polyester—1 bi-layer, (**c**) LbL-treated polyester—5 bi-layers, (**d**) LbL-treated polyester—10 bi-layers and (**e**) LbL-treated polyester—20 bi-layers. Reprinted with permission from [50]. Copyright 2019, Elsevier.

Fang and co-workers [50] exploited the layer-by-layer method for providing polyester fabrics with flame retardant and anti-drippring features. To this end, 1, 5, 10 and 20 bi-layers were deposited onto the synthetic fabric substrate. Limiting Oxygen Index and vertical flame spread tests revealed the very good performances of the fabrics treated with 10 and 20 bi-layered architectures, which were found to prevent melt dripping phenomena and achieved self-extinction as well (Figure 20).

**Figure 21:**
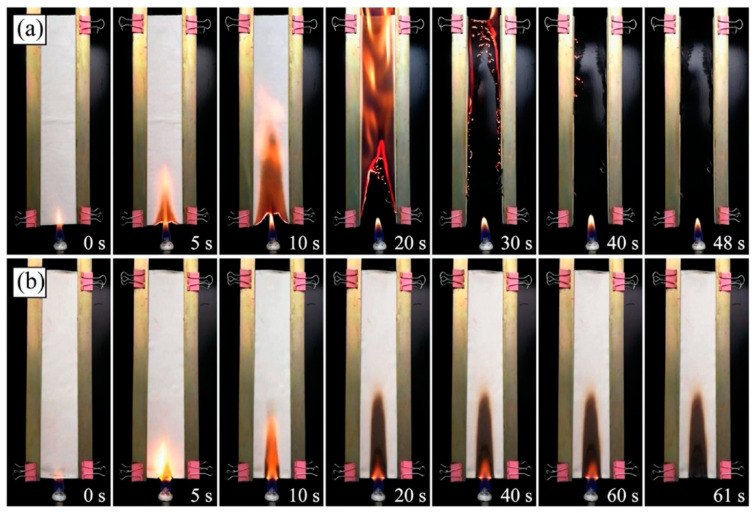
Vertical burning test processes of (**a**) untreated cotton fabric and (**b**) cotton fabric coated with the system containing 50 wt. % of modified montmorillonite in the carboxymethyl chitosan/montmorillonite treating bath, with four repetitions of the dip-coating process. Reprinted with permission from [51]. Copyright 2019, Elsevier.

**Figure 22:**
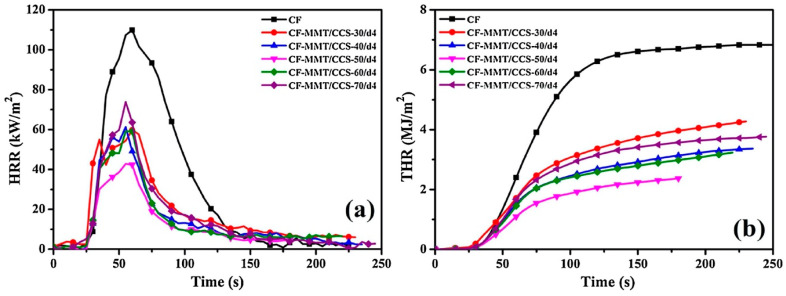
Heat Release Rate (**a**) and Total Heat Release (**b**) curves vs. time for untreated cotton (CF) and for the fabric treated with different modified montmorillonite/carboxymethyl chitosan dip-coating baths. CF-MMT/CCS-*X*/d*n*: *X* represents the weight percent of modified montmorillonite (MMT) in the montmorillonite/carboxymethyl chitosan (CCS) dispersion, while *n* corresponds to the repetition times of the dip-coating process. Reprinted with permission from [51]. Copyright 2019, Elsevier.

Recently, a hybrid organic–inorganic nacre-inspired one-step coating made of carboxymethyl chitosan (carboxylation degree: 83.4%) and an epoxy-silane modified montmorillonite was successfully applied to cotton fabrics by Xie and co-workers [51]. To this end, a dip-coating technique was employed: different fabric specimens were obtained, according to the composition (w. %) of the modified montmorillonite/carboxymethyl chitosan dispersion and to the repetition times of the dip-coating process. The resulting coatings remarkably improved the flame retardant behavior of the treated cellulosic substrate: in particular, the treated fabrics achieved self-extinction in vertical flame spread tests (an example is shown in Figure 21 for the system containing 50 wt. % of modified montmorillonite in the carboxymethyl chitosan/montmorillonite treating bath, with four repetitions of the dip-coating process). Besides, this forced combustion tests revealed a significant decrease in the peak of the Heat Release Rate, the Total Heat Release and the Total Smoke Production values, as well as a remarkable increase (up to 72.4%) in the residues at the end of the tests (Figure 22).

**Figure 23:**
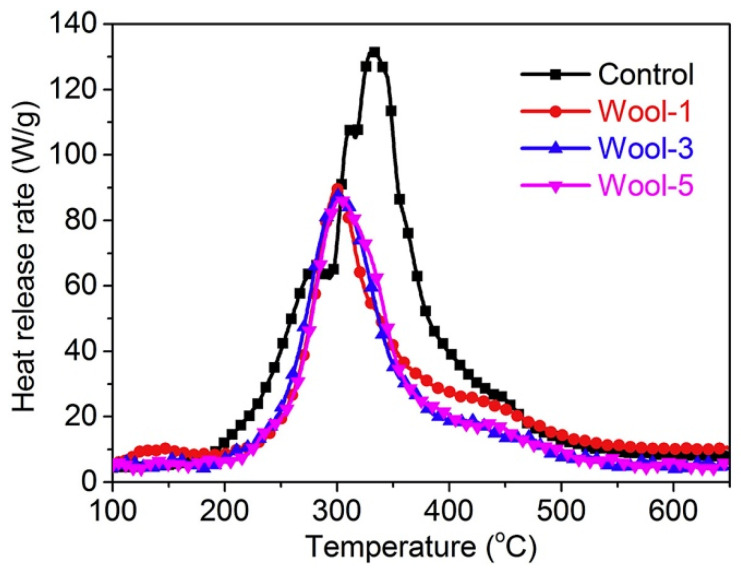
Heat Release Rate vs. temperature for untreated wool (“Control”) and for the fabric treated with the polyelectrolyte complex with different deposit times. Reprinted with permission from [52]. Copyright 2019, Elsevier.

Cheng and co-workers designed and applied a water-soluble bio-resourced polyelectrolyte complex made of phytic acid and chitosan to wool fabrics, utilizing citric acid either as a solvent for chitosan or as a crosslinker [52]. Three different deposit times (namely, 1, 3 and 5) were adopted, thus progressively increasing the final dry add-on on the protein substrate (9.1%, 15.1% and 19.8%, respectively). The so-treated fabrics achieved self-extinction in vertical flame spread tests. Besides this, it is worthy of note that deposit time 5 maintained the self-extinguishing character of the treated fabrics, even after 10 washing cycles. Finally, as revealed by pyrolysis-combustion flow calorimetry tests, the proposed polyelectrolyte complex was found to significantly decrease the peak of the Heat Release Rate and the Total Heat Release (Figure 23), increasing, at the same time, the residues at the end of the tests (from 15.9% to 29.0% for untreated wool and for the fabric with the highest add-on, respectively).

## 5. Chitosan and Its Derivatives as Flame Retardants for Foams or for Wood

As compared to bulky polymers and textiles, the use of chitosan and its derivatives for conferring flame retardant properties to foamed materials or to wood is quite limited. The main achievements will be summarized in the following.

**Figure 24:**
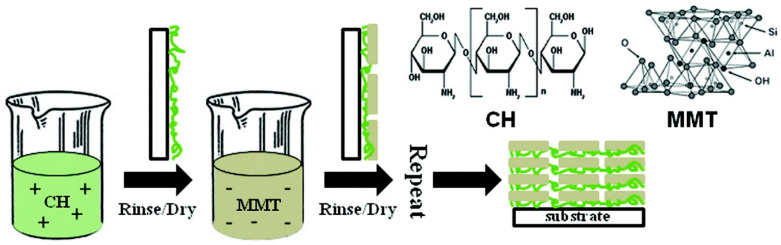
Schematic representation of Layer-by-Layer (LbL) assembly with chitosan (CH) and sodium montmorillonite clay (MMT). This process is repeated until the desired number of bilayers is deposited. Reprinted with permission from [53]. Copyright 2012, American Chemical Society.

The first papers dealing with the use of chitosan and/or its derivatives for designing flame retardant foams date back to 8–10 years ago. One of the pioneering works, by Laufer and co-workers [53], investigated the combination of positively charged chitosan at two different pH levels (namely, 3 and 6) with anionic sodium montmorillonite, giving rise to the formation of bi-layered assemblies (namely, 10, 15, 20, 25 and 30 bi-layers) on the surface of a flexible polyurethane foam, according to the scheme displayed in Figure 24.

**Figure 25:**
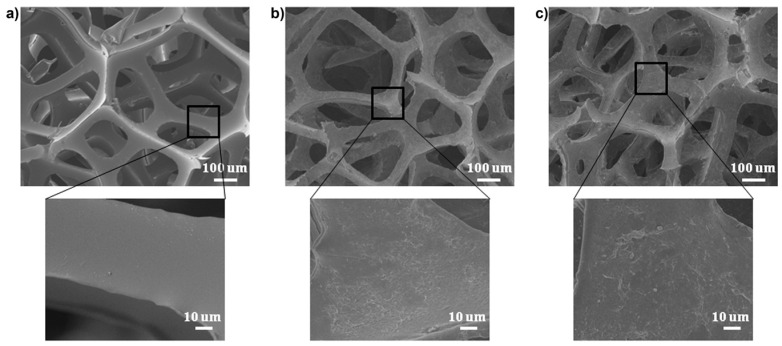
Scanning Electron Microscopy (SEM) images of (**a**) uncoated polyurethane foam; (**b**,**c**) foam coated with 10 bi-layers of chitosan/sodium montmorillonite at pH = 3 (panel (**b**)); foam coated with 10 bi-layers of chitosan/sodium montmorillonite at pH = 6 (panel (**c**)). Reprinted with permission from [53]. Copyright 2012, American Chemical Society.

The morphology of the LbL-treated foams was investigated through SEM analyses (Figure 25): it is worthy of note that unlike the untreated foam, which showed a very smooth surface, the coated counterparts displayed a homogeneous nanotexture that confirmed the conformal nature of the Layer-by-Layer assembly. In particular, every pore wall of the foam was well covered by the LbL architecture, without any change in the macroscale porosity of the coated substrate.

**Figure 26:**
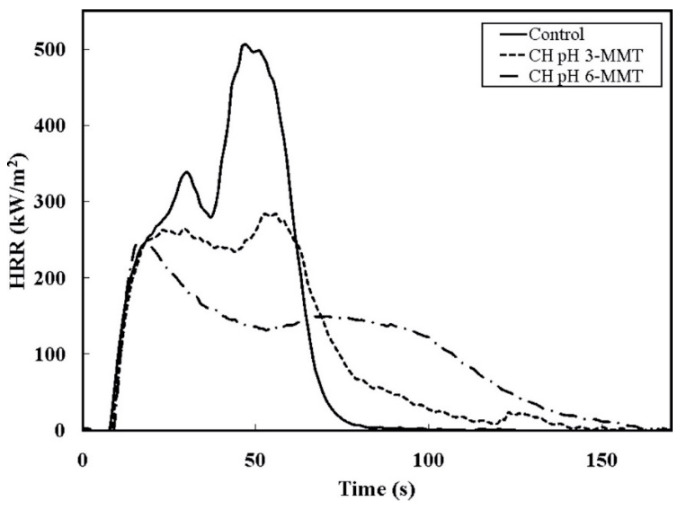
Heat-Release Rate (HRR) as a function of time during cone calorimeter testing for control (uncoated polyurethane foam) and for the foams coated with 10 bi-layers of chitosan/sodium montmorillonite at pH = 3 (CH pH 3-MMT) and at pH = 6 (CH pH 6-MMT). Reprinted with permission from [53]. Copyright 2012, American Chemical Society.

The incorporation of just 10 bi-layers, irrespective of the pH at which they were assembled, turned out to be sufficient for preventing the melting of the foam when directly exposed to the flame from a butane torch. Besides, as assessed by cone calorimetry tests, the same assemblies promoted a remarkable decrease in the peak of the Heat Release Rate (by about 52%) with respect to the untreated substrate (Figure 26).

Pursuing this research, the same group replaced sodium montmorillonite with vermiculite nanoplatelets, obtaining results similar to [53] and demonstrating a very high thermal shielding effect exerted by the deposited assemblies [54].

**Figure 27:**
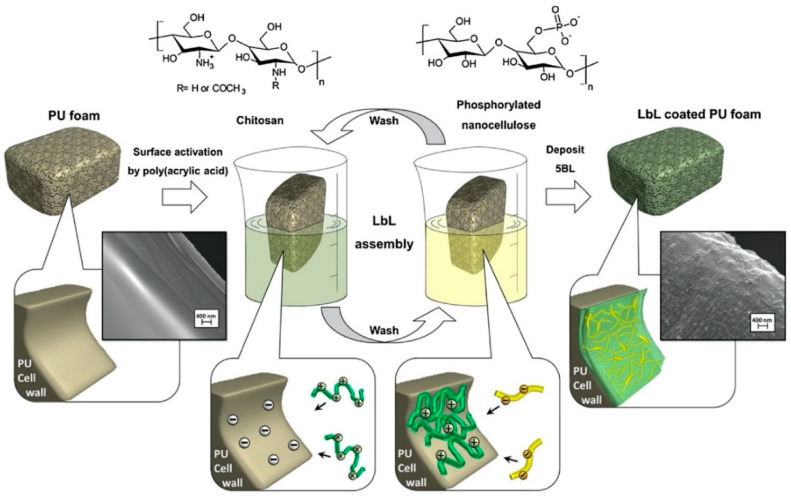
Schematic representation of the LbL process. Polyurethane foams were pre-activated by poly(acrylic acid) and then alternatively dipped in the chitosan (positive) and phosphorylated cellulose nanofibrils (negative) baths. The process was repeated 5 times in order to deposit 5 bi-layers. Reprinted with permission from [55]. Copyright 2018, Elsevier.

**Figure 28:**
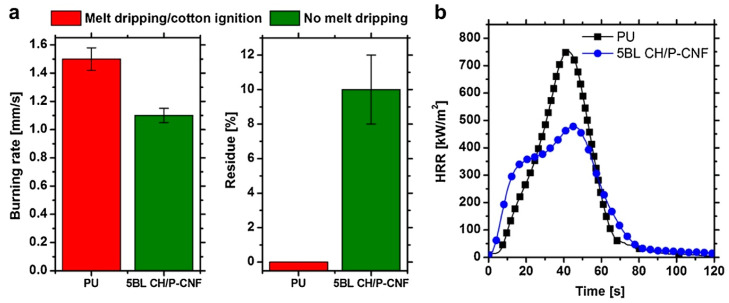
Burning rates and final residues evaluated by flame spread tests performed in horizontal configuration (**a**) and Heat Release Rate (HRR) plots as a function of time measured by cone calorimetry tests (**b**). PU: untreated polyurethane foam; 5 BL CH/P-CNF: foam treated with 5 bi-layers of chitosan and phosphorylated cellulose nanofibrils. Reprinted with permission from [55]. Copyright 2018, Elsevier.

Quite recently, phosphorylated cellulose nanofibrils were coupled with chitosan using the Layer-by-Layer method and applied to a flexible polyurethane foam [55], according to the scheme displayed in Figure 27. In particular, five deposited bi-layers (corresponding to 8% weight gain) were enough to prevent melt dripping phenomena during horizontal flame spread tests, reducing, at the same time, the peak of the Heat Release Rate by 31%, as revealed by cone calorimetry tests (Figure 28).

**Figure 29:**
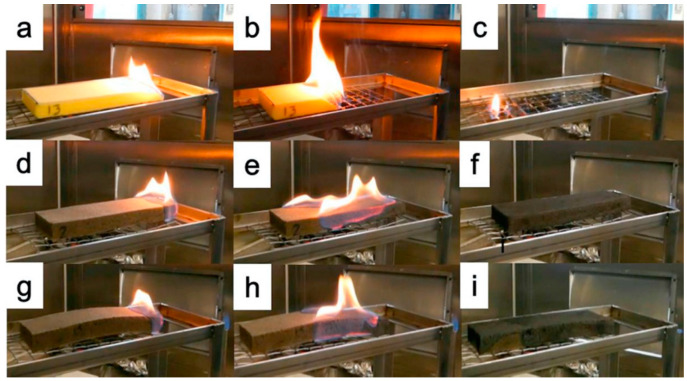
Pictures of horizontal flame spread tests of untreated polyurethane foam (**a**–**c**), foam coated with 3 chitosan/graphene oxide bi-layers (**d**–**f**) and foam coated with 6 chitosan/graphene oxide bi-layers (**g**–**i**). First column, right after ignition; second column, 15 s after ignition; third column, during flame out. Reprinted with permission from [56]. Copyright 2018, Elsevier.

**Figure 30:**
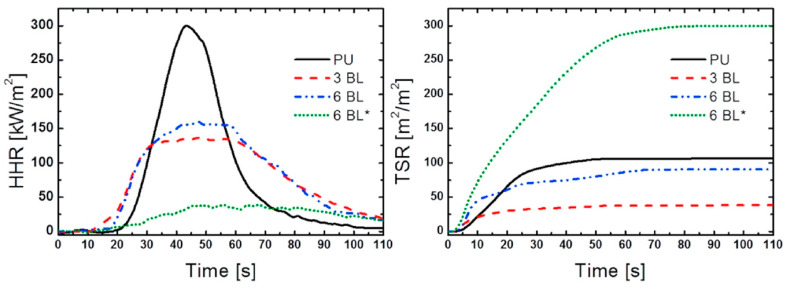
Heat Release Rate (**a**) and Total Smoke Release (TSR) curves of untreated polyurethane foam (PU) and of foams coated with 3 (“3BL”) and 6 (“6BL”) chitosan/graphene oxide bi-layers. 6BL* indicates non-igniting samples. Reprinted with permission from [56]. Copyright 2018, Elsevier.

Then, the same group replaced phosphorylated cellulose nanofibrils with graphene oxide (GO) platelets, thus depositing three and six bi-layered assemblies on a flexible polyurethane foam [56]. As revealed by flame spread tests carried out in a horizontal configuration, the deposition of only three bi-layers suppressed the melt dripping phenomena (Figure 29); during forced-combustion tests, this assembly significantly decreased both the peak of the Heat Release Rate (−54%) and the Total Smoke Release (−59%) values (Figure 30). Conversely, half of the specimens treated with six bi-layers did not ignite when exposed to 35 kW/m^2^ irradiative heat flux. These findings highlight the effectiveness of the designed assemblies, wherein GO platelets, which act either as reinforcing agents for the polyurethane foam or slow down the release of volatile combustible species, are embedded in the aromatic stable char generated by chitosan.

**Figure 31:**
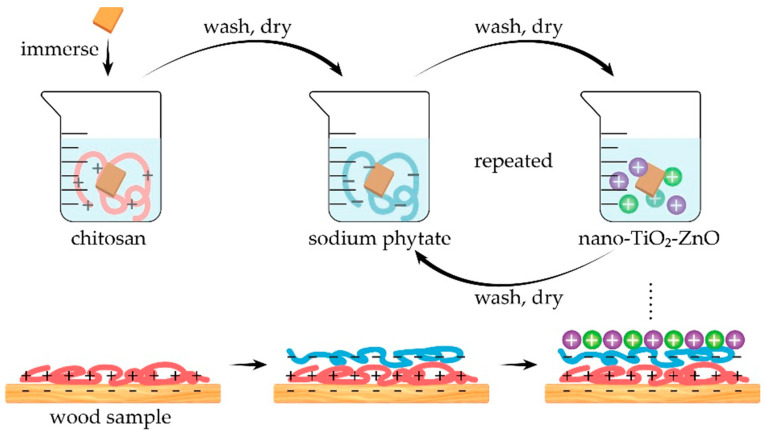
Scheme of the Layer-by-Layer process. Reprinted from [57] under CC BY 4.0 license.

To the best of the authors’ knowledge, the scientific literature reports only one very recent paper dealing with the use of chitosan as a component of flame retardant recipes for wood. In particular, Zhou and Fu [57] exploited the Layer-by-Layer technique for depositing assemblies made of chitosan, sodium phytate and TiO_2_-ZnO nanoparticles, according to the scheme shown in Figure 31. In order to achieve a homogeneous coverage of the underlying substrate, 10 deposition cycles were performed, and for comparison, aiming at investigating the effect of a single component of the LbL architectures, wood was also coated with assemblies made of (i) chitosan, (ii) chitosan and sodium phytate, (iii) chitosan, sodium phytate and nano-TiO_2_, or (iv) chitosan, sodium phytate and nano-ZnO.

**Figure 32:**
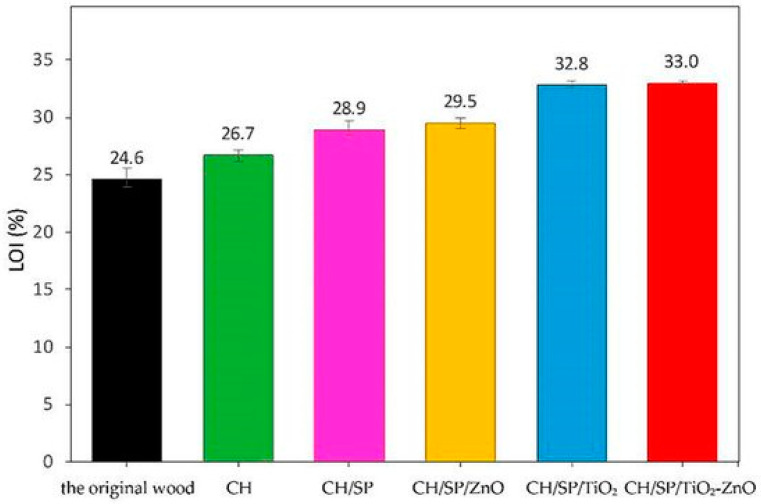
Limiting Oxygen Index values for uncoated and LbL-treated wood. CH: Chitosan-coated (green); CH/SP: chitosan sodium phytate-coated (purple); CH/SP/nano-ZnO: chitosan/sodium phytate/nano-ZnO-coated (yellow); CH/SP/nano-TiO_2_: chitosan/sodium phytate/nano-TiO_2_-coated (blue); CH/SP/nano-TiO_2_-ZnO: chitosan/sodium phytate/nano-TiO_2_-ZnO-coated. Reprinted from [57] under CC BY 4.0 license.

The flame retardant properties of all the samples were assessed by means of Limiting Oxygen Index tests. As shown in Figure 32, the LOI values increased as the composition increased, reaching 33% when wood was coated with the assembly comprising all the LbL constituents.

**Figure 33:**
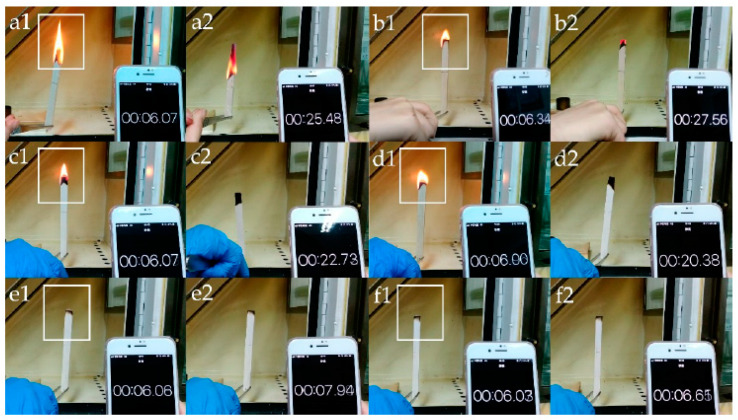
Propensity for flame burning of wood and the coated samples: (**a1**,**a2**) original wood; (**b1**,**b2**) chitosan-coated sample; (**c1**,**c2**) chitosan sodium phytate-coated sample; (**d1**,**d2**) chitosan/sodium phytate/nano-ZnO-coated sample; (**e1**,**e2**) chitosan/sodium phytate/nano-TiO_2_-coated sample; (**f1**,**f2**) chitosan/sodium phytate/nano-TiO_2_-ZnO-coated sample. Reprinted from [57] under CC BY 4.0 license.

To further support these flammability data, wood and the LbL-coated systems underwent direct exposure to an alcohol lamp flame. Flame initiation, spreading and self-extinguishing time were recorded for the samples treated with the different components; the results are displayed in Figure 33. In particular, it was found that:

— the burning flame of the coated wood samples was much smaller than that of the original wood within 6 s of ignition;
— the fire of the sample coated with chitosan only extended slightly from the ignition point. In fact, the chitosan layer was scarcely effective in providing wood with flame retardance, as already witnessed by the Limiting Oxygen Index values (Figure 32);
— the flame retardant properties were enhanced after the addition of the sodium phytate layer, which, upon the application of the flame, converted into phosphoric acid, thus favoring the formation of a stable char through dehydration reactions;
— finally, upon the introduction of TiO_2_ and/or ZnO nanoparticle layers, the wood required a relatively shorter time of 6–20 s to extinguish the flame, hence confirming the thermal shielding effect provided by the selected inorganic systems.

## 6. Conclusions and Future Perspectives

The world of flame retardance is currently experiencing a new inclination toward the design, development and application of flame retardant products with a low environmental impact and an efficiency that, in many cases, is almost comparable to that of traditional chemical FR additives.

In this context, bio(macro)molecules and bio-sourced products are gathering much interest, at present mainly from academic researchers. In fact, several papers dealing with the use of these low environmental impact products have been published so far, clearly demonstrating, at least at a lab scale, their great potentialities. In the near future, continuous growth of the research work on this specific topic is expected, notwithstanding that this activity matches well with the circular economy concept, as some of the proposed bio(macro)molecules and bio-sourced products/extracts can be derived from wastes, by-products or crops, hence providing a new added value to end of life resources.

The “green character” of chitosan, which has already been reported in the literature [58,59], together with the multifunctionality that this biomacromolecule can provide to polymeric materials, fully justify its current use in different application sectors; besides, its chemical structure and composition have suggested an unexpected applicability in the field of flame retardance, aiming at designing new flame retardant formulations suitable for different polymer substrates (namely, bulky polymers, fabrics, foams and wood). In particular, chitosan has started to be considered a valuable carbon source, usually in combination with other flame retardant additives that may effectively interact with chitosan, thus enhancing the overall FR performance; indeed, the possibility of its conversion into a stable (aromatic) protective char makes this biomacromolecule very appealing.

As shown in the review, chitosan can be easily chemically modified, mainly by introducing phosphorus-based groups that can synergistically interact with the carbon source upon exposure to a flame or to an irradiative heat source. The so-obtained chitosan derivatives have also shown a good potential for the design of effective flame retardant systems.

Despite the great potentialities provided by chitosan and its derivatives, some challenging issues are still currently under discussion.

First of all, in the context of a low environmental impact approach, it is quite difficult to design a flame retardant recipe that is fully bio-sourced, i.e., combining chitosan (or its derivatives, whose green character may be questionable because of the chemistry behind their synthesis) with other biomacromolecules or bio-products. Some successful attempts have been made so far, but further efforts still have to be carried out.

Another up-to-date issue refers to the durability of the chitosan-based flame retardant recipes, especially when chitosan and/or its derivatives are employed as surface treatments (as for the buildup of LbL assemblies). Indeed, due to the waterborne character of components of the layers, the washing speed of these coatings is somehow limited if their recipe does not include specific crosslinkers. In this context, as described in the present work, some partially successful attempts have been performed, by either exploiting the reactivity of chitosan to UV-radiation (i.e., the propensity of chitosan for self-crosslinking) or the addition of such cross-linkers as genipin and citric acid, among a other things. The solution of this challenging issue is therefore still quite far from being fully achieved, and new green strategies, involving natural and effective cross-linking systems, have to be found and developed.

In conclusion, further advances in the design and development of low environmental impact FRs based on chitosan and its derivatives can be foreseen for the very near future, paving the way to increased sustainability.

## Figures and Tables

**Table 1:** Main chemical modifications of chitosan for flame retardant purposes.

Substrate	Chemical Modification of Chitosan	Ref.
Thermoplastic polyurethane	Chitosan crosslinked with bis-(4-formylphenyl)-phenyl-phosphonate	[29]
Thermoplastic polyurethane	Phosphorylated chitosan	[30]
Linear low density polyethylene	Melamine salt of chitosan phosphate	[31]
Thermoplastic polyurethane	Toluene diisocyanate cross-linked carboxymethyl chitosan microencapsulating melamine polyphosphate	[32]

**Table 2:** Results from cone calorimetry tests.

Sample	Time to Ignition (s)	Peak of Heat Release Rate (kW/m^2^)	Δ(%)	Total Heat Release (MJ/m^2^)
PA66	28	256	-	11.2
PA66 “one pot” UV-cured	20	306	+19.5	9.5
PA66 “LbL” UV-cured	12	191	−25.4	10.6

**Table 3:** Flame retardant behavior of untreated cotton and the treated fabrics.

Sample	Final Dry Add-on (%)	Limiting Oxygen Index (%)	Time to Ignition (s)	Peak of Heat Release Rate (kW/m^2^)	Final Residue (%)
Cotton	-	18	14	181	0.1
Cotton + chitosan	8.04	21	13	162	2.1
Cotton + ammonium phytate	7.98	29	8	92	16.9
Cotton LbL treated with chitosan and ammonium phytate	8.00	27	7	80	20.1

## References

[1] Troitzsch J. (1990). International Plastics Flammability Handbook.

[2] Horrocks A.R., Price D. (2001). Fire Retardant Materials.

[3] Gaan S., Salimova V., Rupper P., Ritter A., Schmid H., Pan N., Sun G. (2011). Flame retardant functional textiles. Functional Textiles for Improved Performance, Protection and Health.

[4] Hofer H. (1998). Health Aspects of Flame Retardants in Textiles.

[5] Horrocks A.R., Price D. (2006). Fire Retardant Materials.

[6] Horrocks A.R., Anand S.C., Hill B.J. (1997). Fire and Heat Resistant Materials. U.S. Patent.

[7] Alongi J., Malucelli G., Tiwari A., Raj B. (2015). Reactions and Mechanisms in Thermal Analysis of Advanced Materials.

[8] Lewin M., Le Bras M., Camino G., Bourbigot S., Delobel R. (1998). Physical and Chemical Mechanisms of Flame Retarding of Polymers. Fire Retardancy of Polymers—The Use of Intumescence.

[9] Aslin D.C. (1989). The design and development of intumescent coatings for structural fire protection. J. Oil Colour Chem. Assoc..

[10] Camino G., Costa L., Luda M.P. (1993). Mechanistic aspects of intumescent fire retardant systems. Makromol. Chem. Makromol. Symp..

[11] Le Bras M., Bourbigot S., Revel B. (1999). Comprehensive study of the degradation of an intumescent EVA-based material during combustion. J. Mater. Sci..

[12] Alongi J., Han Z., Bourbigot S. (2015). Intumescence: Tradition vs. novelty. A comprehensive review. Prog. Polym. Sci..

[13] Gann R.G., Dipert R.A., Drews M.J. (1987). Encyclopedia of Polymer Science and Engineering.

[14] Camino G., Costa L. (1988). Performance and mechanisms of fire retardants in polymer—A review. Polym. Degrad. Stab..

[15] Mark H.F., Atlas S.M., Shalaby S.W., Pearce E.M., Lewin M., Atlas S.M., Pearce E.M. (1975). Combustion of polymers and its retardation. Flame-Retardant Polymeric Materials.

[16] Van der Veen I., De Boer J. (2012). Phosphorus Flame Retardants: Properties, Production, Environmental Occurrence, Toxicity and Analysis. Chemosphere.

[17] Zaikov G.E., Lomakin S.M. (2002). Ecological Issue of Polymer Flame Retardancy. J. Appl. Polym. Sci..

[18] Hull T.R., Law R.J. (2014). Environmental Drivers for Replacement of Halogenated Flame Retardants.

[19] Salmeia K.A., Gaan S., Malucelli G. (2016). Recent Advances for Flame Retardancy of Textiles Based on Phosphorus Chemistry. Polymers.

[20] Yang T.T., Guan J.P., Tang R.C., Chen G. (2018). Condensed tannin from Dioscorea cirrhosa tuber as an eco-friendly and durable flame retardant for silk textile. Ind. Crop. Prod..

[21] Basak S., Samanta K.K., Chattopadhyay S.K., Narkar R.S., Mahangade R. (2015). Flame retardant cellulosic textile using banana pseudostem sap. Int. J. Cloth. Sci. Tech..

[22] Basak S., Wazed Ali S. (2017). Leveraging flame retardant efficacy of pomegranate rind extract, a novel biomolecule, on ligno-cellulosic materials. Polym. Degrad. Stab..

[23] Kumar Dutta P., Dutta J., Tripathi V.S. (2004). Chitin and chitosan: Chemistry, properties and applications. J. Sci. Ind. Res..

[24] Shirvan A.R., Shakeri M., Bashari A. (2019). Recent Advances in Application of Chitosan and its Derivatives in Functional Finishing of Textiles in The Impact and Prospects of Green Chemistry for Textile Technology.

[25] Rinaudo M. (2006). Chitin and chitosan: Properties and applications. Prog. Polym. Sci..

[26] Majeti N.V., Kumar R. (2000). A review of chitin and chitosan applications. React. Funct. Polym..

[27] Younes I., Rinaudo M. (2015). Chitin and Chitosan Preparation from Marine Sources. Structure, Properties and Applications. Mar. Drugs.

[28] Corazzari I., Nisticò R., Turci F., Faga M.G., Franzoso F., Tabasso S., Magnacca G. (2015). Advanced physico-chemical characterization of chitosan by means of TGA coupled on-line with FTIR and GCMS: Thermal degradation and water adsorption capacity. Polym. Degrad. Stab..

[29] Zhang S., Liua X., Jin X., Li H., Sun J., Gu X. (2018). The novel application of chitosan: Effects of cross-linked chitosan on the fire performance of thermoplastic polyurethane. Carbohydr. Polym..

[30] Liu X., Guo J., Tang W., Li H., Gu X., Sun J., Zhang S. (2019). Enhancing the flame retardancy of thermoplastic polyurethane by introducing montmorillonite nanosheets modified with phosphorylated chitosan. Compos. Part. A.

[31] Hassan M., Nour M., Abdelmonem Y., Makhlouf G., Abdelkhalik A. (2016). Synergistic effect of chitosan-based flame retardant and modified clay on the flammability properties of LLDPE. Polym. Degrad. Stab..

[32] Liu X., Sun J., Zhang S., Guo J., Tang W., Li H., Gu X. (2019). Effects of carboxymethyl chitosan microencapsulated melamine polyphosphate on the flame retardancy and water resistance of thermoplastic polyurethane. Polym. Degrad. Stab..

[33] Chen Z., Jiang J., Yu Y., Zhang Q., Chen T., Ni L. (2020). Layer-by-layer assembled diatomite based on chitosan and ammonium polyphosphate to increase the fire safety of unsaturated polyester resins. Powder Technol..

[34] Richardson J.J., Cui J., Björnmalm M., Braunger J.A., Ejima H., Caruso F. (2016). Innovation in layer-by-layer assembly. Chem. Rev..

[35] Zhang X., Xu Y., Zhang X., Wu H., Shen J., Chen R., Xiong Y., Li J., Guo S. (2019). Progress on the layer-by-layer assembly of multilayered polymer composites: Strategy, structural control and applications. Prog. Polym. Sci..

[36] Prabhakar M.N., Song L. (2018). Fabrication and characterisation of starch/chitosan/flax fabric green flame-retardant composites. Int. J. Biol. Macromol..

[37] Rao T.N., Hussain I., Koo B.H. (2020). Enhanced thermal properties of silica nanoparticles and chitosan bio-based intumescent flame retardant Polyurethane coatings. Mater. Today Proc..

[38] Jiao C., Li M., Chen X., Li S. (2020). Flame retardancy and thermal decomposition behavior of TPU/chitosan composites. Polym. Adv. Technol..

[39] Pan H., Wang W., Pan Y., Song L., Hu Y., Liew K.M. (2015). Formation of self-extinguishing flame retardant biobased coating on cotton fabrics via Layer-by-Layer assembly of chitin derivatives. Carbohydr. Polym..

[40] Liu Y., Wang Q., Jiang Z., Zhang C., Li Z., Chen H., Zhu P. (2018). Effect of chitosan on the fire retardancy and thermal degradation properties of coated cotton fabrics with sodium phytate and APTES by LBL assembly. J. Anal. Appl. Pyrol..

[41] Sheikh J., Bramhecha I. (2018). Multifunctional modification of linen fabric using chitosan based formulations. Int. J. Biol. Macromol..

[42] Zhang Z., Ma Z., Leng Q., Wang Y. (2019). Eco-friendly flame retardant coating deposited on cotton fabrics from bio-based chitosan, phytic acid and divalent metal ions. Int. J. Biol. Macromol..

[43] Lv Z., Hu Y., Guan J., Tang R., Chen G. (2019). Preparation of a flame retardant, antibacterial, and colored silk fabric with chitosan and vitamin B2 sodium phosphate by electrostatic layer by layer assembly. Mater. Lett..

[44] Jordanov I., Magovac E., Fahami A., Lazar S., Kolibaba T., Smith R.J., Bischof S., Grunlan J.C. (2019). Flame retardant polyester fabric from nitrogen-rich low molecular weight additives within intumescent nanocoating. Polym. Degrad. Stabil..

[45] El-Tahlawy K. (2008). Chitosan phosphate: A new way for production of eco-friendly flame retardant cotton textiles. J. Text. I.

[46] Kumar Kundu C., Wang X., Hou Y., Hu Y. (2018). Construction of flame retardant coating on polyamide 6.6 via UV grafting of phosphorylated chitosan and sol–gel process of organo-silane. Carbohydr. Polym..

[47] Kumar Kundu C., Wang X., Song L., Hu Y. (2020). Chitosan-based flame retardant coatings for polyamide 66 textiles: One-pot deposition versus layer-by-layer assembly. Int. J. Biol. Macromol..

[48] Pan Y., Liu L., Zhang Y., Song L., Hu Y., Jiang S., Zhao H. (2019). Effect of genipin crosslinked layer-by-layer self-assembled coating on the thermal stability, flammability and wash durability of cotton fabric. Carbohydr. Polym..

[49] Li P., Wang B., Liu Y., Xu Y., Jiang Z., Dong C., Zhang L., Liu Y., Zhu P. (2020). Fully bio-based coating from chitosan and phytate for fire-safety and antibacterial cotton fabrics. Carbohydr. Polym..

[50] Fang Y., Liu X., Tao X. (2019). Intumescent flame retardant and anti-dripping of PET fabrics through layer-by-layer assembly of chitosan and ammonium polyphosphate. Prog. Org. Coat..

[51] Xie H., Lai X., Wang Y., Li H., Zeng X. (2019). A green approach to fabricating nacre-inspired nanocoating for superefficiently fire-safe polymers via one-step self-assembly. J. Hazard. Mater..

[52] Cheng X., Guan J., Yang X., Tang R., Yao F. (2019). A bio-resourced phytic acid/chitosan polyelectrolyte complex for the flame retardant treatment of wool fabric. J. Clean Prod..

[53] Laufer G., Kirkland C., Cain A.A., Grunlan J.C. (2012). Clay−Chitosan Nanobrick Walls: Completely Renewable Gas Barrier and Flame-Retardant Nanocoatings. ACS Appl. Mater. Interfaces.

[54] Lazar S., Carosio F., Davesne A., Jimenez M., Bourbigot S., Grunlan J.C. (2018). Extreme Heat Shielding of Clay/Chitosan Nanobrick Wall on Flexible Foam. ACS Appl. Mater. Interfaces.

[55] Carosio F., Ghanadpour M., Alongi M., Wågberg L. (2018). Layer-by-layer-assembled chitosan/phosphorylated cellulose nanofibrils as a bio-based and flame protecting nano-exoskeleton on PU foams. Carbohydr. Polym..

[56] Maddalena L., Carosio F., Gomez J., Saracco G., Fina A. (2018). Layer-by-layer assembly of efficient flame retardant coatings based on high aspect ratio graphene oxide and chitosan capable of preventing ignition of PU foam. Polym. Degrad. Stabil..

[57] Zhou L., Fu Y. (2020). Flame-Retardant Wood Composites Based on Immobilizing with Chitosan/Sodium Phytate/Nano-TiO2-ZnO Coatings via Layer-by-Layer Self-Assembly. Coatings.

[58] Muñoz I., Rodríguez C., Gillet D., Moerschbacher B.M. (2018). Life cycle assessment of chitosan production in India and Europe. Int. J. Life Cycle Ass..

[59] Leceta I., Guerrero P., Cabezudo S., De La Caba K. (2013). Environmental assessment of chitosan-based films. J. Clean Prod..

